# Reverse pneumatic artificial muscles (rPAMs): Modeling, integration, and control

**DOI:** 10.1371/journal.pone.0204637

**Published:** 2018-10-12

**Authors:** Erik H. Skorina, Ming Luo, Wut Yee Oo, Weijia Tao, Fuchen Chen, Sina Youssefian, Nima Rahbar, Cagdas D. Onal

**Affiliations:** 1 Robotics Engineering Program, Worcester Polytechnic Institute, Worcester, MA 01609, United States of America; 2 Department of Mechanical Engineering, Stanford University, Stanford, CA 94305. United States of America; 3 Department of Mechanical Engineering, Worcester Polytechnic Institute, Worcester, MA 01609, United States of America; 4 Department of Civil Engineering, Worcester Polytechnic Institute, Worcester, MA 01609, United States of America; UCLA, UNITED STATES

## Abstract

Despite offering many advantages over traditional rigid actuators, soft pneumatic actuators suffer from a lack of comprehensive, computationally efficient models and precise embedded control schemes without bulky flow-control valves and extensive computer hardware. In this article, we consider an inexpensive and reliable soft linear actuator, called the reverse pneumatic artificial muscle (rPAM), which consists of silicone rubber that is radially constrained by symmetrical double-helix threading. We describe analytical and numerical static models of this actuator, and compare their performance against experimental results. To study the application of rPAMs to operate underlying kinematic linkage skeletons, we consider a single degree-of-freedom revolute joint that is driven antagonistically by two of these actuators. An analytical model is then derived, and its accuracy in predicting the static joint angle as a function of input pressures is presented. Using this analytical model, we perform dynamic characterization of this system. Finally, we propose a sliding-mode controller, and a sliding mode controller augmented by a feed-forward term to modulate miniature solenoid valves that control air flow to each actuator. Experiments show that both controllers function well, while the feed-forward term improves the performance of the controller following dynamic trajectories.

## 1 Introduction

Due to their weight and rigidity, robots operated by traditional motors can be dangerous to humans, limiting their ability to operate efficiently in human-trafficked environments. Soft actuators can absorb energy to enable safe and compliant physical interaction with the environment in a way that is similar to biological muscles, allowing for a bio-inspired approach to robotics and actuation. This paper describes a soft linear actuation concept inspired by biological anatomy we call the *reverse pneumatic artificial muscle* (rPAM), which we use to apply forces on underlying kinematic chains. This actuator is called the rPAM because it operates on similar principles to the traditional PAM (also known as the McKibben actuator [1]), only with a reversed direction of actuation (similar to the work of [2]). Specifically, our approach utilizes pre-strained fiber-reinforced elastomer tubes that relieve contractile stresses upon pressurization, hence offering stable antagonistic forces on either side of revolute joints with minimal radial deformation.

The rPAM is an example of a Fiber Reinforced Elastomeric Enclosure (FREE). The first example of this is the McKibben muscle which contracts under pressurization due to the geometry of an external braided mesh [1, 3]. McKibben actuators use fluidic power to offer large forces and fast responses in a way that is analogous to biological muscles [4] but may suffer from significant radial expansion as well as modeling and control difficulties. [5] showed that a large deformation membrane-model with two families of inextensible fibers accurately predicts the static response of McKibben actuators. McKibben actuators can be connected together in either series or parallel to achieve more complex movements [6].

A different type of FREE was invented in [2] and recently studied by Bishop-Moser, Krishnan and Kota [7, 8]. This soft actuator consists of a hollow cylinder of silicone rubber with fibers wrapped around the outside. The relative inextensibility of the external fibers with respect to the silicone creates geometric constraints on the deformation of the actuator when pressurized. Depending on the exact geometry of the fiber reinforcements, they can cause the actuator to undergo axial expansion, contraction, bending, or twisting when pressurized. Bishop-Moser, Krishnan and Kota [7, 8] explore the mobility characteristics of FREEs with double-helix fiber configurations, both symmetrical and asymmetrical. This work has also been extended to FREEs with double helical fibers and an additional thread in order to achieve the mobility needed for snake-like soft robots [9]. These differ from McKibben actuators in the nature of the fiber reinforcement, which is a braided mesh for McKibben actuators but a simpler composition of helical fibers for this newer work.

**Fig 1:**
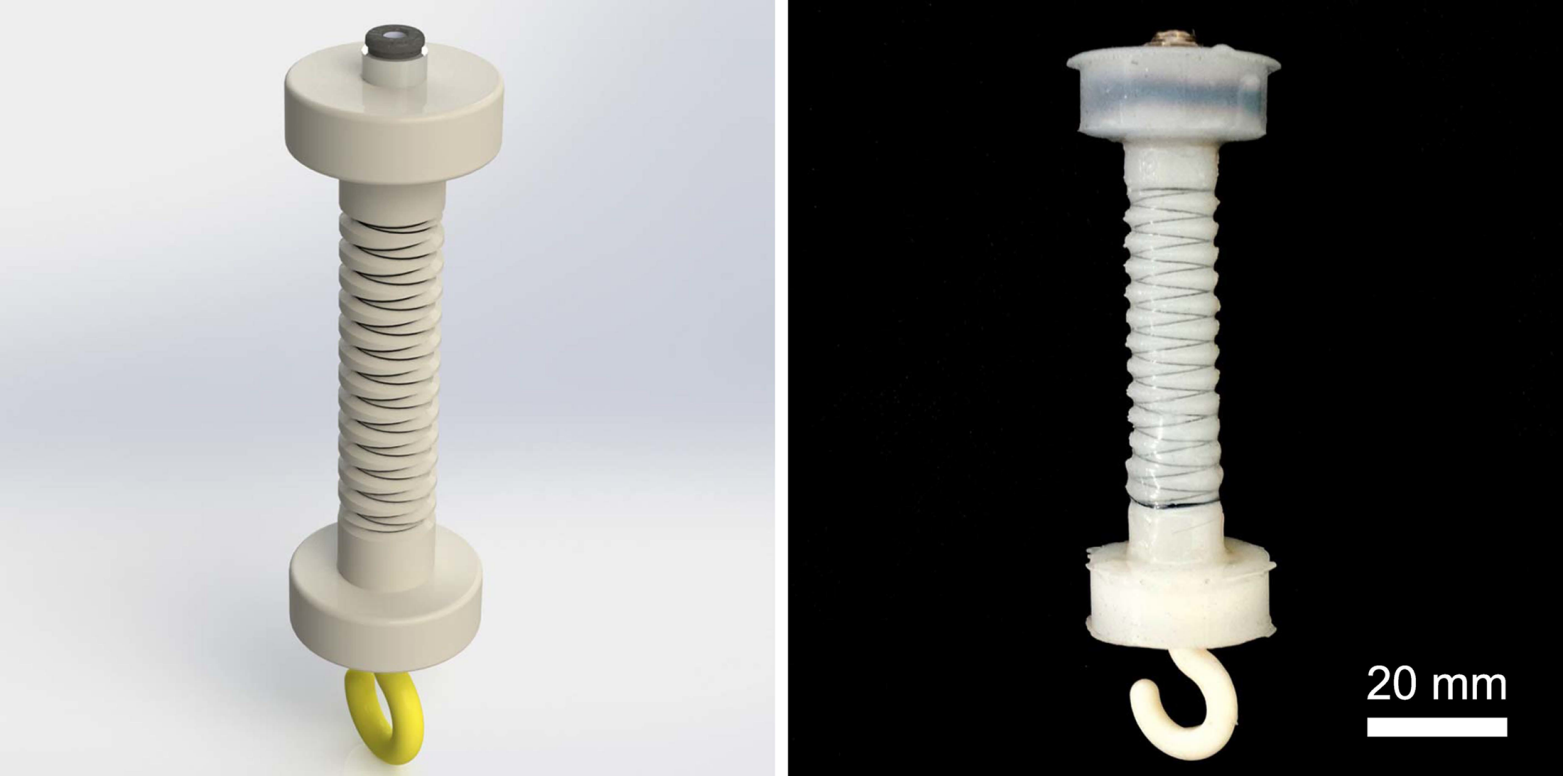
A CAD model (left) and a physical prototype (right) of the proposed soft linear actuator, called reverse pneumatic artificial muscle (rPAM), which offers convenient physical and fluidic connectors to operate rigid kinematic linkages.

For linear muscle-like soft actuation and the ability to exert larger forces, this paper considers a single cylindrical pressure chamber reinforced radially by fibers wound in two symmetrical small-angle helices to approximate a series of circles along the length, as an extensile version of McKibben actuators. Thus, hoop stresses in the composite are opposed by the inextensible thread while the axial stresses lead to deformation. The resulting actuators are inexpensive, easier and faster to fabricate and can provide large forces due to a larger range of input pressures (than fluidic elastomer actuators used in our earlier work [10–15], and other similar articles in the recent literature [16–19]). Soft linear actuation is not very useful for extension motion outputs, as it can easily buckle under a payload. Our solution to this challenge is to operate the fiber-reinforced soft actuators *in reverse* to achieve rPAMs, by not applying vacuum, but ensuring that the actuator always encounters tensile stresses. We achieve this by pre-straining the actuator and releasing the corresponding stresses through pressurization. Fig 1 shows a picture of the proposed soft linear actuators.

A modeling approach to these actuators has previously been shown using conservation of energy and the geometrical constraints created by fiber enclosures [8, 20, 21]. In addition, [22] expanded upon this model to consider the distortion effects at the termination nodes and the radial pressure loss due to rubber elasticity. However, these analytical models do not cover all of the forces because the material properties of the elastomeric substrate (typically silicone rubber) contribute to the force balance. [23] uses a nonlinear Mooney-Rivlin term to consider the nonlinear elasticity of the bladder in order to reduce the modeling error. However, the Mooney-Rivlin model has limitations for large deformations of rubbers [24]. Therefore, a more accurate model needs to incorporate the properties of silicone rubber to obtain the stored strain energy and to accurately calculate the forces and torques generated by the actuator.

Thus, the objectives of this paper are three-fold: First is to implement and validate analytical and numerical models of the rPAM, which utilize accurate material properties to predict actuator performance. With a reliable analytical model, the performance of a given actuator configuration can be ascertained with minimal experimental work, subsequently allowing the control for kinematic chains operated by rPAMs. The second objective is to apply the developed model to a fundamental practical application: operating a 1-Degree-of-Freedom (DoF) revolute joint. The validation of the developed model in this context would highlight its effectiveness as a tool for predicting actuator performance under realistic use cases. The third objective is to use two different controllers to address the precise motion control problem of the 1-DoF revolute joint. The first one is a sliding mode controller [25], while the second one combines the developed sliding mode controller with a static mapping function to create a feedforward augmented sliding mode (SM+FF) controller. This results in a system that is both faster and more compact that existing soft pneumatic actuation systems, while still being capable of precise control.

The contributions of this work include:

- A computationally efficient and modular analytical model of rPAMs that incorporates the nonlinear material properties of silicone rubber under large strains.
- A finite element analysis model of rPAMs to compare with the analytical model.
- Analytical modeling, dynamic characterization, and control of a 1-DoF revolute joint driven antagonistically by a pair of rPAMs.

We previously discussed the sliding-mode control approach and experimental verification on the 1-DoF revolute joint in [26]. This paper connects our novel advances in soft actuator modeling and verification with feedback control of the integrated soft pneumatic actuated system, providing a complete picture of our advances in soft robotics.

## 2 Reverse pneumatic artificial muscle fabrication, analytical modeling, and verification

### 2.1 Actuator fabrication process

**Fig 2:**
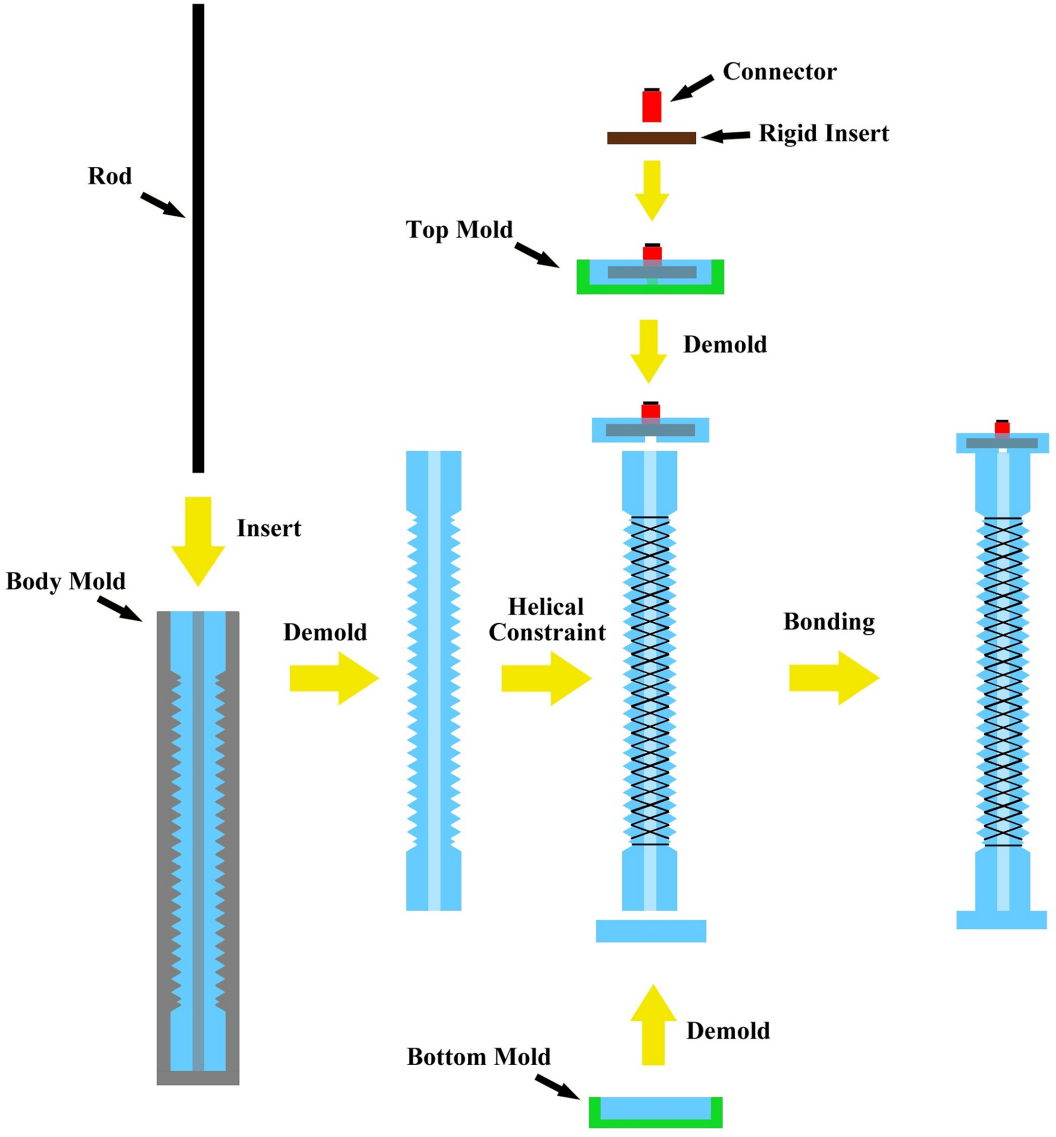
The fabrication process of the proposed rPAM.

The presented rPAM was made by molding silicone rubber (Smooth-on Dragonskin 10) in a 3-D printed mold. The step-by-step fabrication process (shown in Fig 2) is described below:

**Step 1:** Insert a carbon fiber or metallic rod of appropriate diameter into the center of the body mold to create the hollow cylindrical core inside the actuator. Introduce silicone rubber into the body mold with the rod inside, the top connector mold and the bottom mold.
**Step 2:** After silicone rubber has cured, remove the rod inside the body mold, then remove the silicone rubber from the body mold.
**Step 3:** Tie two symmetrical helices of thread around the cylindrical silicone rubber body guided by the grooves. Apply a thin layer of uncured silicone rubber on the threads to immobilize the thread around the actuator.
**Step 4:** Embed the pneumatic fitting connector into the top mold before the silicone rubber had fully cured. Once cured, remove the top and bottom silicone rubber pieces from their respective molds. Bond these to the silicone rubber body using a thin layer of uncured silicone rubber.

### 2.2 Analytical model

**Fig 3:**
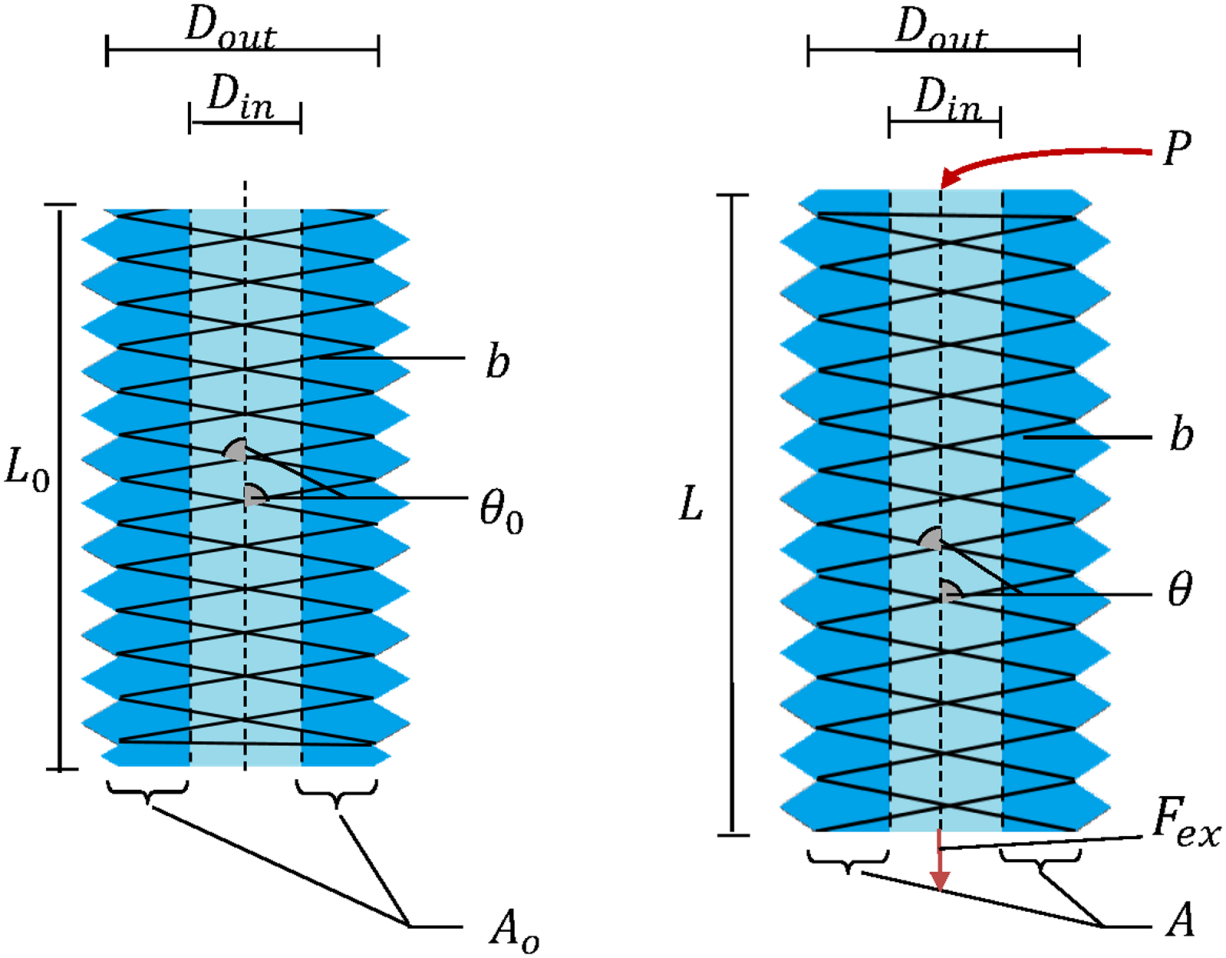
Geometric parameters of the rPAM before (left) and after deformation (right).

We have developed a static analytical model of our soft pneumatic actuators. This model predicts the extension of the actuator under a given internal pressure and load. Our model consists of two components: the constraint model and the material model. The constraint model takes into account the geometrical relationships of the helical threads while the material model takes into account the material properties of the silicone used for the actuator using the Ogden material model. Table 1 and Fig 3 show the parameters used in the analytical model. For simplicity, we make the following standard assumptions about the system.

Assumption 1. There is no shift of or friction from the thread when actuated. This is ensured by the fact that the threads are slotted into grooves and then glued in place.
Assumption 2. The actuator remains cylindrical.
Assumption 3. The thread is inextensible.
Assumption 4. The silicone rubber material is incompressible.
Assumption 5. The analytical model will be quasi-static, describing the position of the actuator at steady state. All experimental data was taken at steady state as well.

**Table 1:** Parameters of the soft actuator model.

Symbol	Description
*P*	Input air pressure
*V*	Soft actuator volume
*n*	The number of helical turns of the thread
*A*	The nominal material cross sectional area
*A*_*o*_	The initial nominal cross sectional area
*A*_*in*_	The internal cross sectional area
*θ*	Helix angle
*L*_*o*_	Initial length of the soft actuator
*L*	Length of the soft actuator after deformation
*b*	Total thread length
*D*_*out*_	Outer diameter of the soft actuator
*D*_*in*_	Inner diameter of the soft actuator
*σ*	The axial stress within the material
λ	The principal stretch
*F*_*cons*_	Helical constraint force
*F*_*int*_	Force due to internal material deformation
*F*_*ext*_	External force

The analytical model of rPAMs is based on force balance in steady state as:

$$\begin{array}{c} F_{ext}=F_{cons}+F_{int}, \end{array}$$ (1)

where *F*_*ext*_ is the external axial load on the actuator in tension, *F*_*cons*_ is the helical constraint force and *F*_*int*_ is the force due to internal material stresses calculated based on the Ogden model.

To develop the model for the helical constraint force, we consider calculating an effective internal area based on the McKibben actuator analytical model [8, 20, 27], also taking the effect of wall thickness into account. As the pneumatic actuator is a cylinder, its external volume can be calculated as follows:

$$\begin{array}{c} V_{out}=\frac{\pi D_{out}^{2}}{4}L. \end{array}$$ (2)

where $\frac{\pi D_{out^{2}}}{4}$ is the cross sectional area of the actuator and *L* is its length, as discussed in Table 1. From here, we can use trigonometric relations to calculate *V*_*out*_ in terms of *b*, *θ*, and *n*. From Fig 3, we can deduce that $D_{out}=\frac{bsin(\theta )}{\pi n}$ and *L* = *bcos*(*θ*). Thus, substituting in, we get:

$$V_{out}=\frac{b^{3}sin^{2}\theta cos\theta }{4\pi n^{2}}$$ (3)

Input pressure acts on the internal cross sectional area. Thus, it is necessary for us to calculate the internal cross sectional area. First, it can be put as:

$$A_{in}=A_{out}-A$$ (4)

where *A*_*out*_ is cross section of the entire actuator, and *A* is the cross sectional area of the actuator wall. We can substitute in using the work in [27] to get:

$$\begin{array}{c} \begin{array}{cc} A_{in}= & \frac{dV_{out}}{dL}-\frac{A_{o}}{\lambda }\\ = & \frac{b^{2}-3L^{2}}{4\pi n^{2}}-\frac{A_{o}}{\lambda }, \end{array} \end{array}$$ (5)

where the initial cross sectional area $A_{o}=\frac{\pi (D_{out}^{2}-D_{in}^{2})}{4}$ and the principal stretch $\lambda =\frac{L}{L_{o}}=\frac{A_{o}}{A}$ for incompressible material [28]. Thus, the helical constraint force can be calculated as:

$$\begin{array}{c} F_{cons}=-PA_{in}=-P(\frac{b^{2}-3L^{2}}{4\pi n^{2}}-\frac{A_{o}}{\lambda }) \end{array}$$ (6)

The next step is to determine the internal material force *F*_*int*_. To model the internal forces within the actuator, we turned to Ogden hyperelastic solid model [29], which is a powerful tool to analytically describe the deformation of a broad range of elastomeric materials assuming that the nonlinear stress-strain relationship in the material can be described using a strain energy density function [30–32]. Using this model, the axial stress [31] follows the form:

$$\begin{array}{c} \sigma =\sum_{i=1}^{3}\frac{2\mu_{i}}{\alpha_{i}}\left(\lambda^{\alpha_{i}}-\lambda^{-\frac{\alpha_{i}}{2}}\right), \end{array}$$ (7)

for three Ogden elements, where *μ*_*i*_ and *α*_*i*_ are material constants obtained by experimental tensile testing data. Consequently, the internal forces due to material deformation can be written as:

$$\begin{array}{c} \begin{array}{cc} F_{int} & =\sigma A\\ & =\sum_{i=1}^{3}\frac{2\mu_{i}A_{o}}{\alpha_{i}}\left(\lambda^{\alpha_{i}-1}-\lambda^{-\frac{\alpha_{i}}{2}-1}\right). \end{array} \end{array}$$ (8)

**Fig 4:**
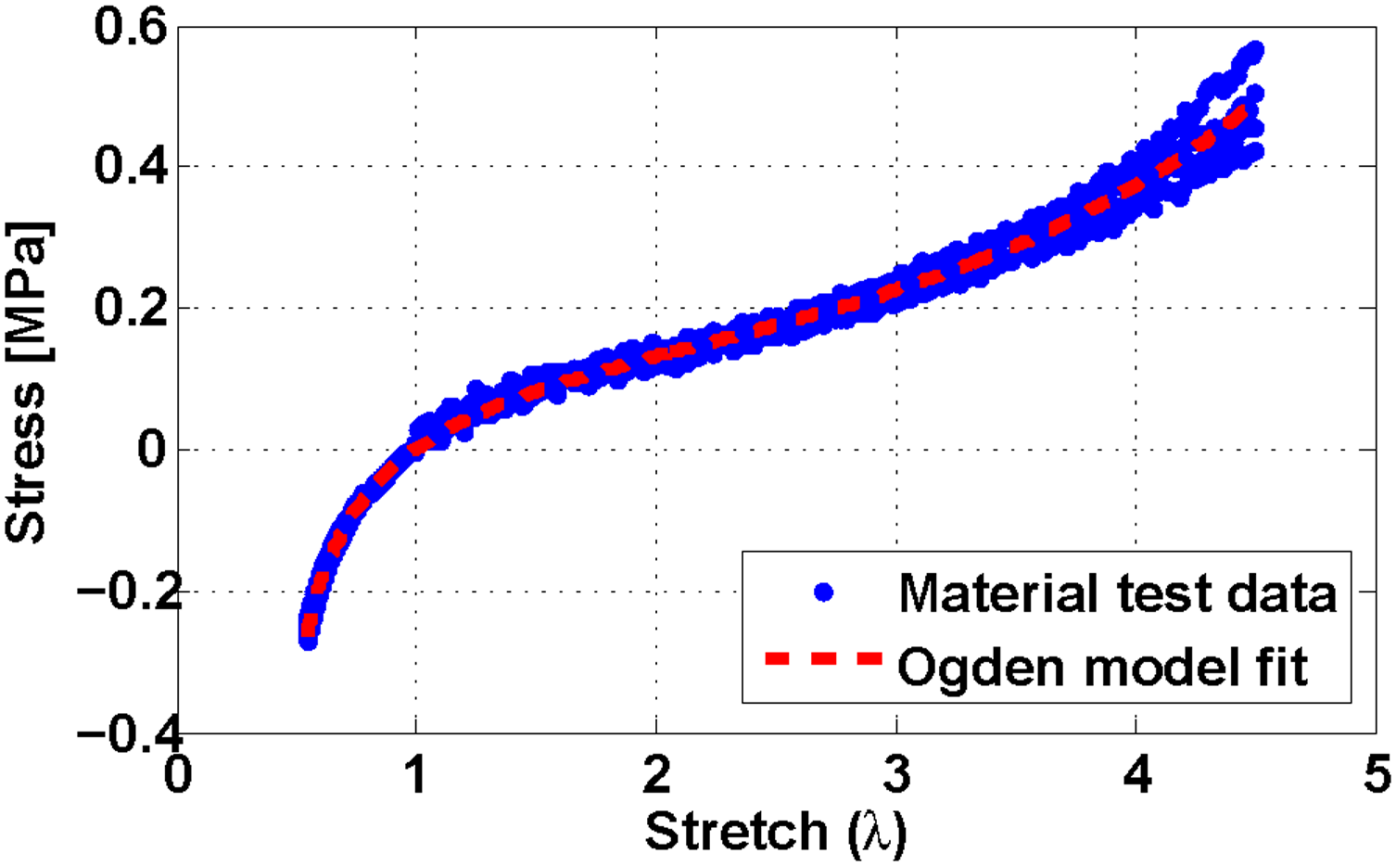
Static stress-strain response of Dragonskin 10 silicone rubber. Blue dots are experimental data points from multiple compressive and tensile material loading tests, while the red dashed line is the Ogden model curve fit. The mean error for this Ogden fit is 1.1 ± 15 kPa.

To calculate the Ogden parameters for our silicone rubber formulation, we performed tension [33] and compression tests [34]. We combined the resulting data and used it to calculate parameters for Eq (7). Fig 4 shows the nonlinear fit curve and Table 2 shows the calculated Ogden parameters.

**Table 2:** Parameter values of the Ogden model.

*μ*_1_	*μ*_2_	*μ*_3_	*α*_1_	*α*_2_	*α*_3_
80031.09	1049.90	32.89	1.62	5.18	-13.42

Together, these components create our analytical model, which can calculate the force output of the actuator at a given length. For static behavior, such as that tested in 2.4, we can also solve the equation for the actuator length that will result in an equilibrium.

### 2.3 Numerical model

Finite element analysis (FEA) is a common tool for modeling soft actuator behavior because of its ability to deal with geometric complexities. To this end we modeled rPAM behavior using Abaqus CAE to provide a numerical solution to support and compare with our analytical one. Our model uses axisymmetry to approximate the full 3-D object. In order to factor in the limitations due to helical threading, we set the outside edge of the actuator to be constrained in the horizontal direction. Thus, it maintains a constant radius of 7 mm while allowing the actuator to extend. The lower end is fixed to represent the actuator mount, while the upper end was capped with more of the silicone rubber and left free. Pressure input is applied directly to the inside surfaces of the actuator as a constant uniform pressure, which simulates steady state. The numerical simulation approximates the effect of the helix with a radial constraint because an accurate representation of the complete helical constraint would require a computationally expensive nonlinear geometric relation.

### 2.4 Actuator static deformation response experimental setup

We performed experiments to evaluate the accuracy of the developed analytical and numerical models. The test set up consisted of the rPAM, a pressurized air source, solenoid valves, a valve control system, and an object tracking system. An air nozzle was used as the pressure source, which was driven and filtered by a precise pressure regulator. For testing, pressure inputs within a range of 0-196 kPa (0-20 psi) with increments of 17 kPa (2.5 psi) were given via the pressure regulator. The regulated pressure was then fed to the actuator through solenoid valves. The rPAM to be tested was clamped at the tip from a rigid holder and allowed to freely hang in the vertical direction. Calibrated weights were hung off the end of the actuator from an attached hook (as seen in Fig 1).

A distinctly colored dot was marked at the tip of the soft actuator, tracked by an external camera throughout the experiments. A line of known length was marked next to the actuator as a reference for data analysis in an object tracking software [35]. The deformation of the actuator under different pressures was measured exactly by the tracking system, giving us precise static deformation data. The solenoid valves receive pressure inputs from the pressure regulator. A control system was designed to turn the solenoid valves on and off at specified periods controlled by an Arduino Uno, in serial communication with a workstation running MATLAB. L298N full-bridge drivers were connected to the controller along with a power supply. As all experiments were performed under static conditions after the actuator had reached steady state, the communication delays were not a concern and the data from the tracking system was calculated off-line.

### 2.5 Actuator deformation results

**Fig 5:**
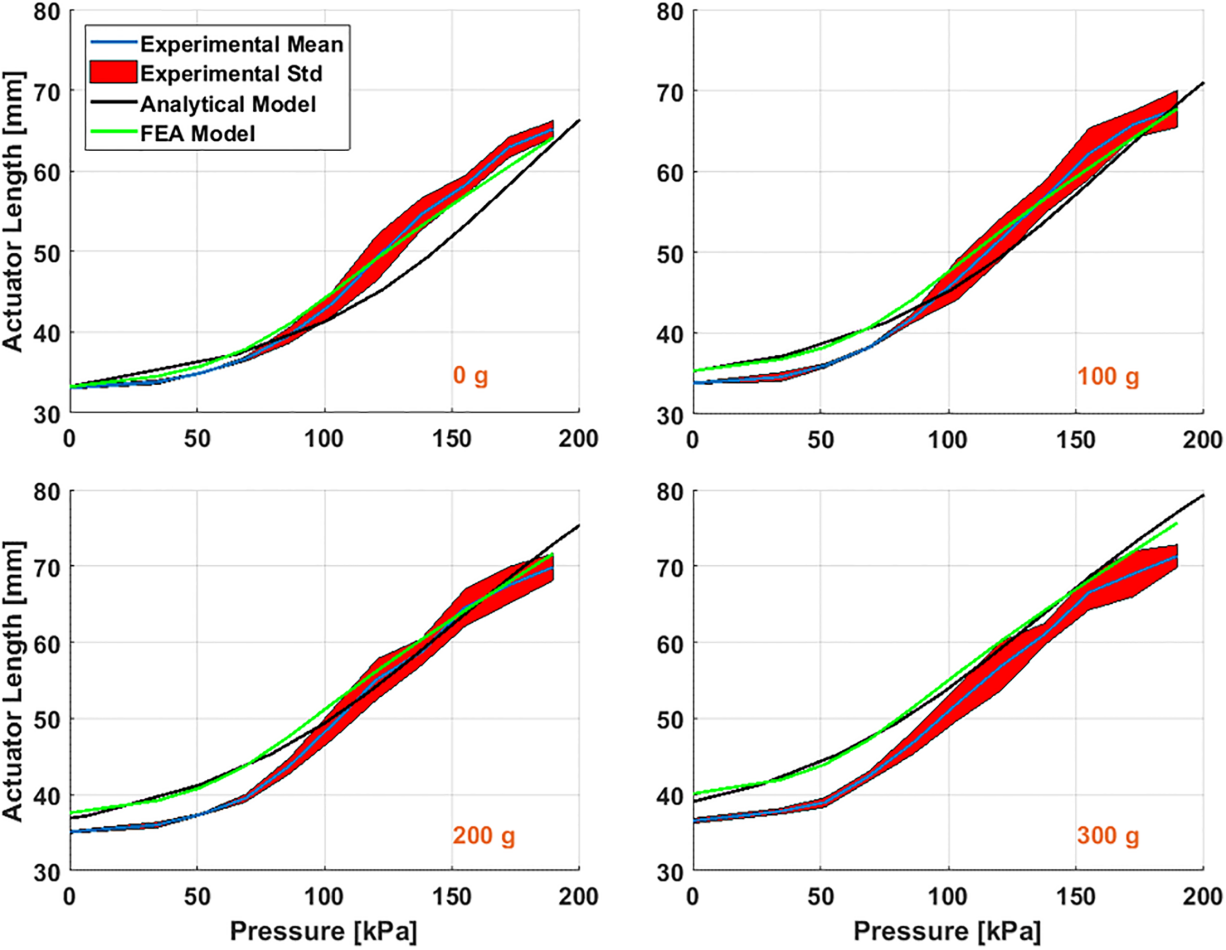
Experimental (mean and standard deviation), analytical, and numerical results for 4 different external loads of 0, 100, 200 and 300 g under input pressures of 0 to 190 kPa.

Table 3 shows the experimental conditions and the parameter values of the rPAM in the experiment. The initial total length of the actuator is 50 mm which consists of primarily the helix-constrained section with a few millimeters of non-helix-constrained section at either end. In order to match the models, we only considered the middle helix constraint part, with an unloaded length of 33 mm. The experiment involved exerting a range of pressures on the actuator while hanging different weights on it. Fig 5 shows a comparison of the static deflection results for each load case.

**Table 3:** Experimental parameters of the rPAM model.

*P*	Input pressure	0-196 kPa
*n*	Number of turns of the thread	8
*L*_*o*_	Initial length of the helix section	33.23 mm
*b*	The total length of the thread	250 mm
*D*_*out*_	The nominal outer diameter	11.54 mm
*D*_*in*_	The inner diameter	5.08 mm
*F*_*ext*_	The external weight	0-300 g

From this figure we can conclude that both models predict similar actuator behavior, with comparable 0 kpa lengths predicted for all 4 weights tested. For 0 g, they diverged at around 70 kpa, with the FEA model having a sharper increase in predicted actuator length than the analytical model. However, at higher weights the FEA model flattened out, more closely matching the analytical model.

At 0g and low pressures (0 kPa to 70 kPa), the models and the experiment match closely. However, at higher pressures the experiment deformed greater than the models, before tapering off near the maximum pressure tested. This could be caused by problems in the material model used, or by deformations of the actuator that only appear at higher pressures (such as stretching of the thread or bulging between the threads). When more weight was added, errors appeared at 0 kPa between the models and the experiment. This was probably the result of the fact that both models don’t incorporate the effects of the threading fully. The FEA model ignored the effects of the thread on actuator behavior (other than keeping the outer diameter constant), while in the analytical model the force from the threading *F*_*cons*_ was dependent on pressure and not on actuator deformation. From Eq (6), when *P* = 0, then *F*_*cons*_ = 0, regardless of how much weight (and therefore deformation) the actuator was undergoing. In reality, an external load will stretch the actuator and deform the helix, which will change the constraint force. However, both models can be useful when predicting actuator behavior at lower pressures and loads. Moreover, since the FEA model does not significantly outperform the analytical model, we do not need to worry about adapting the FEA model for real-time applications. Instead, we can use the analytic model, which is much better suited to our applications of computationally efficient control.

## 3 rPAM-driven revolute joint

**Fig 6:**
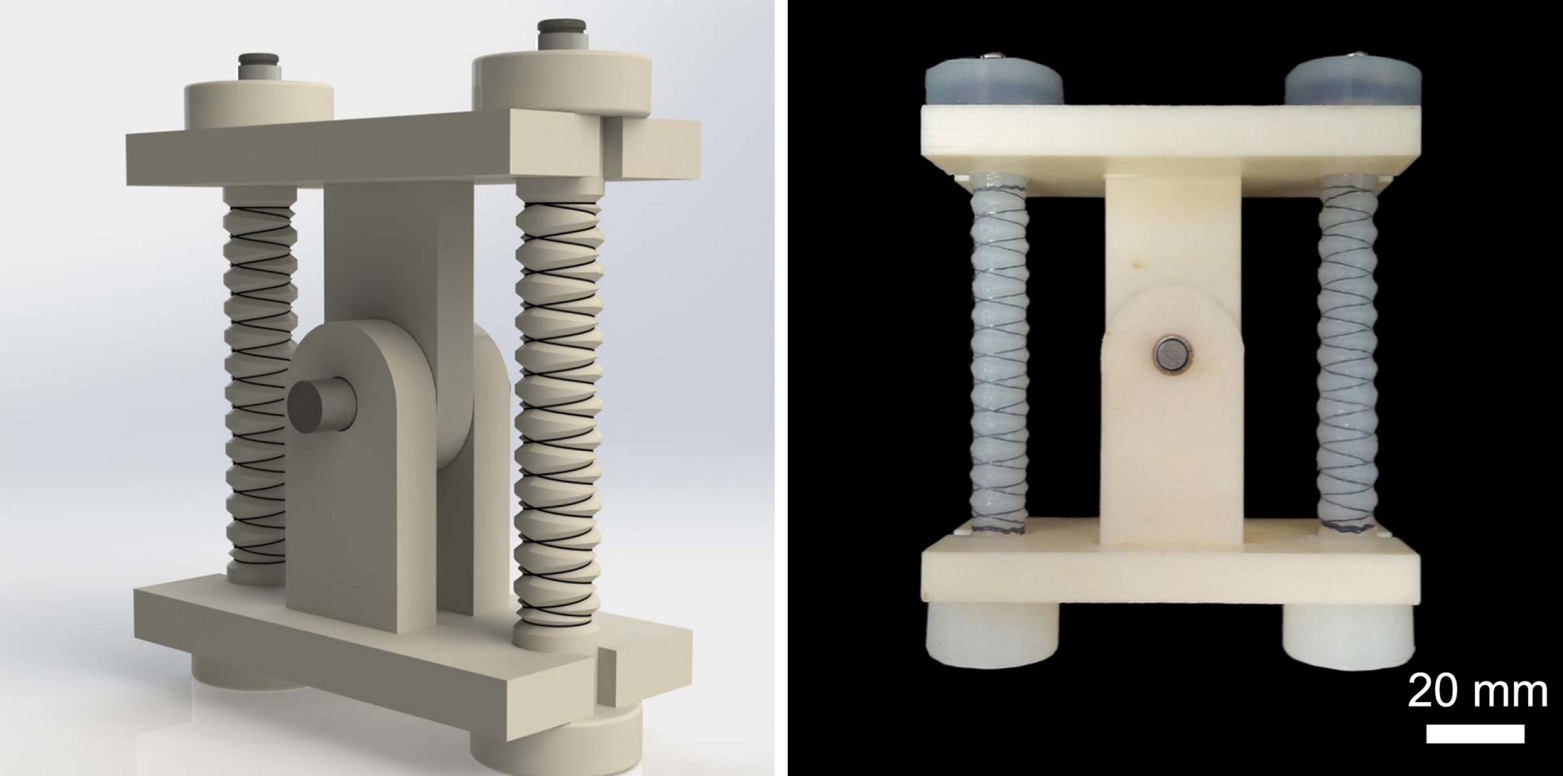
A CAD model (left) and a physical prototype (right) of the 1-DoF revolute joint operated by the proposed rPAMs in antagonism.

To verify our modeling efforts and develop motion control algorithms for soft actuation in a bench-top setting, we designed a simple 1-DoF revolute joint setup, as described previously in [36]. This setup allows us to gauge the rPAM’s usability for driving arbitrary kinematic chains. As the proposed rPAMs extend when pressurized, we use two actuators operating antagonistically to drive our joint, as shown in Fig 6. The joint is designed so that the actuators are both stretched nominally when the joint is at the neutral point. Without this pre-strain keeping the rPAMs under tension, the joint would have to compress one of the rPAMs every time it rotated, forcing that muscle to buckle outwards, and reducing the mobility and usability of the joint. The original length of the threaded component of the actuators is approximately 50 mm, which is stretched to 75 mm at the neutral angle of the joint, for a pre-strain of 50%. In order to perform motion control using a reliable feedback signal, an optical encoder (CUI Inc. AMT 203) was mounted on the joint axis.

### 3.1 Analytical model

In order to calculate the geometric relationship between the joint and the two rPAMs, we assumed that each of the actuators would remain straight during actuation, and apply force directly from the lower mount to the upper mount. This assumption holds up to mid-range pressures, but the actuators were observed to bow under higher pressure differentials and larger joint angles. In the experimental set up, each actuator had an independent pressure regulator. We adjusted each regulator from 41 to 96 kPa in increments of 7 kPa (1 psi) step and recorded the resulting angle for each combination. This range was chosen to maintain the assumption that the rPAMs did not bend or buckle.

**Fig 7:**
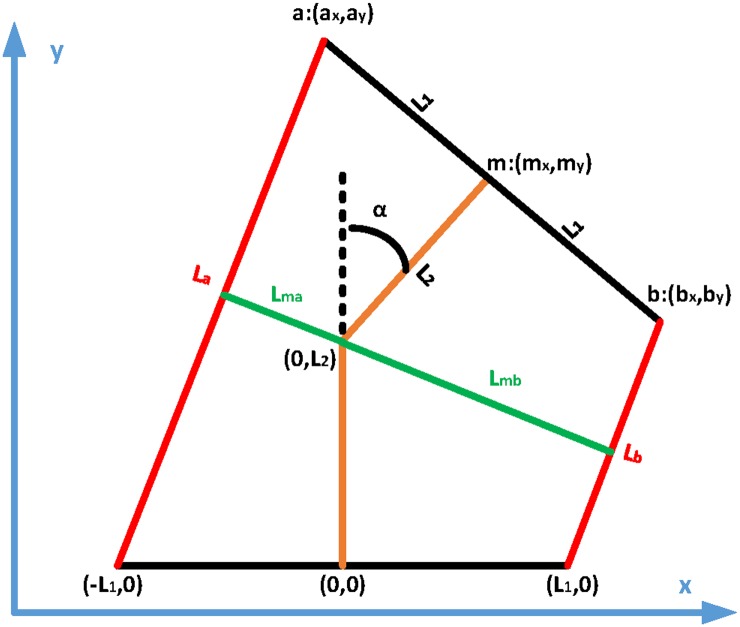
The geometric model of the 1-DoF joint setup. The black and brown lines represent the rigid joint links, while the red lines represent the rPAMs. The green lines are the calculated moment arms for each soft actuator. It should be noted that the actuator is vertically symmetric.

Our analytical model utilized the 1-DoF revolute kinematic model shown in Fig 7. When the joint reaches steady-state, the two external moments exerted by actuators A and B should be equal:

$$\begin{array}{c} \begin{array}{cc} & F_{exta}L_{ma}=F_{extb}L_{mb},\\ & \left(F_{inta}+F_{consa}\right)L_{ma}=\left(F_{intb}+F_{consb}\right)L_{mb}, \end{array} \end{array}$$ (9)

where *L*_*ma*_ and *L*_*mb*_ are the moment arms of the actuators A and B, *L*_*a*_ and *L*_*b*_ are the actuator lengths, and *L*_1_ and *L*_2_ are internal dimensions of the setup. *L*_*a*_ and *L*_*b*_ can be determined as a function of the joint angle *α*, and can be used to calculate *L*_*ma*_ and *L*_*mb*_ as follows:

$$\begin{array}{c} \begin{array}{cc} & L_{ma}=\sqrt{{L_{1}}^{2}+{L_{2}}^{2}-{L_{a}}^{2}/4},\\ & L_{mb}=\sqrt{{L_{1}}^{2}+{L_{2}}^{2}-{L_{b}}^{2}/4}. \end{array} \end{array}$$ (10)

From Eq 9, the joint rotation angle can be determined with respect to input pressures and the proposed analytical model for rPAMs.

**Fig 8:**
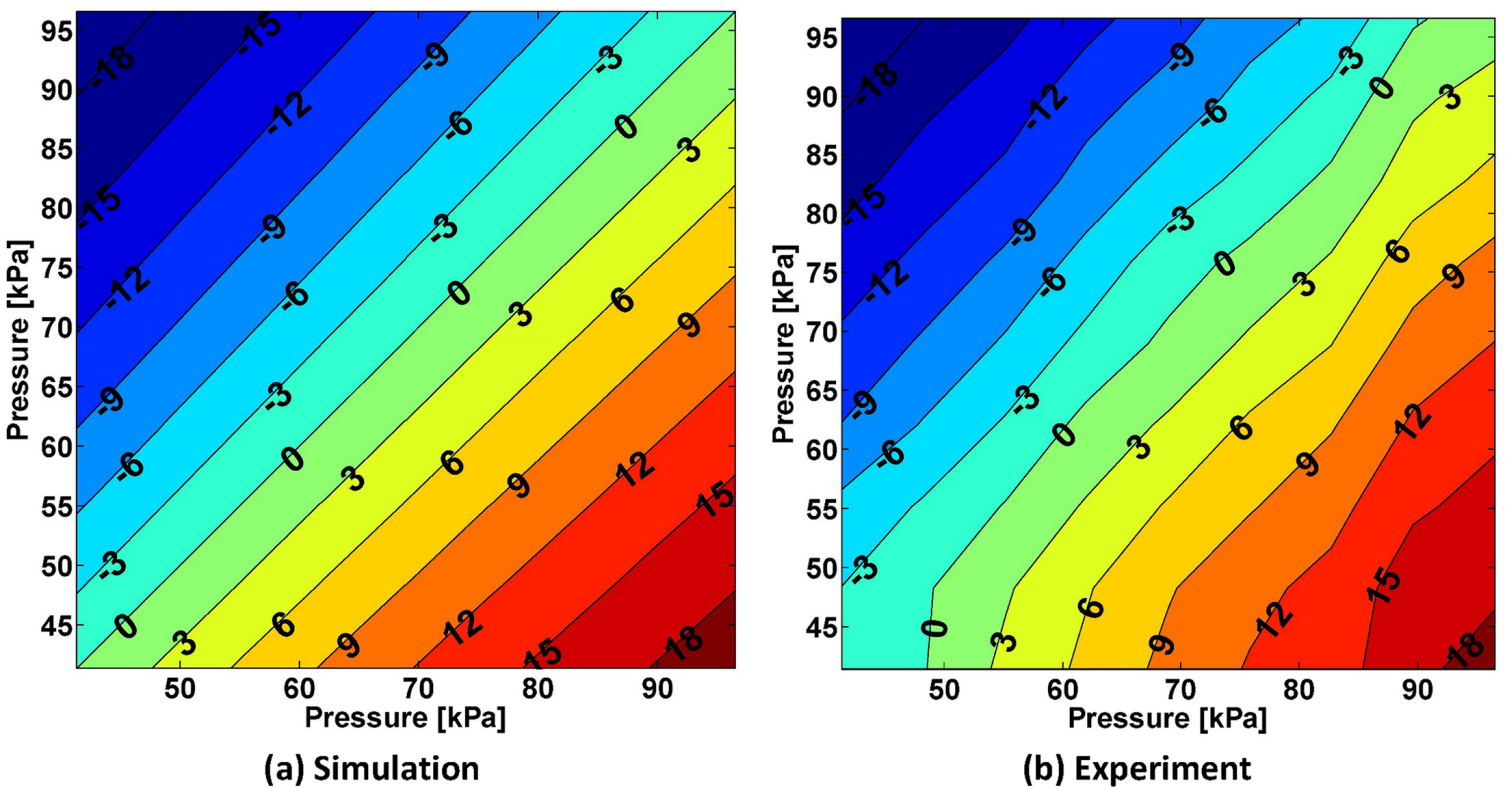
Comparison of model predictions and experimental results for a range of input pressures ranging from 41 to 96 kPa for both rPAMs. Contour plots of the resulting steady-state joint angle in degrees are displayed for simulation (a) and experimental (b) results. Joint angle values are stepped at 3 degrees, annotated on the curves, and indicated as color coding from blue to red. The mean error between the model and the experimental results is 0.27 ± 1.1 degrees.

**Fig 9:**
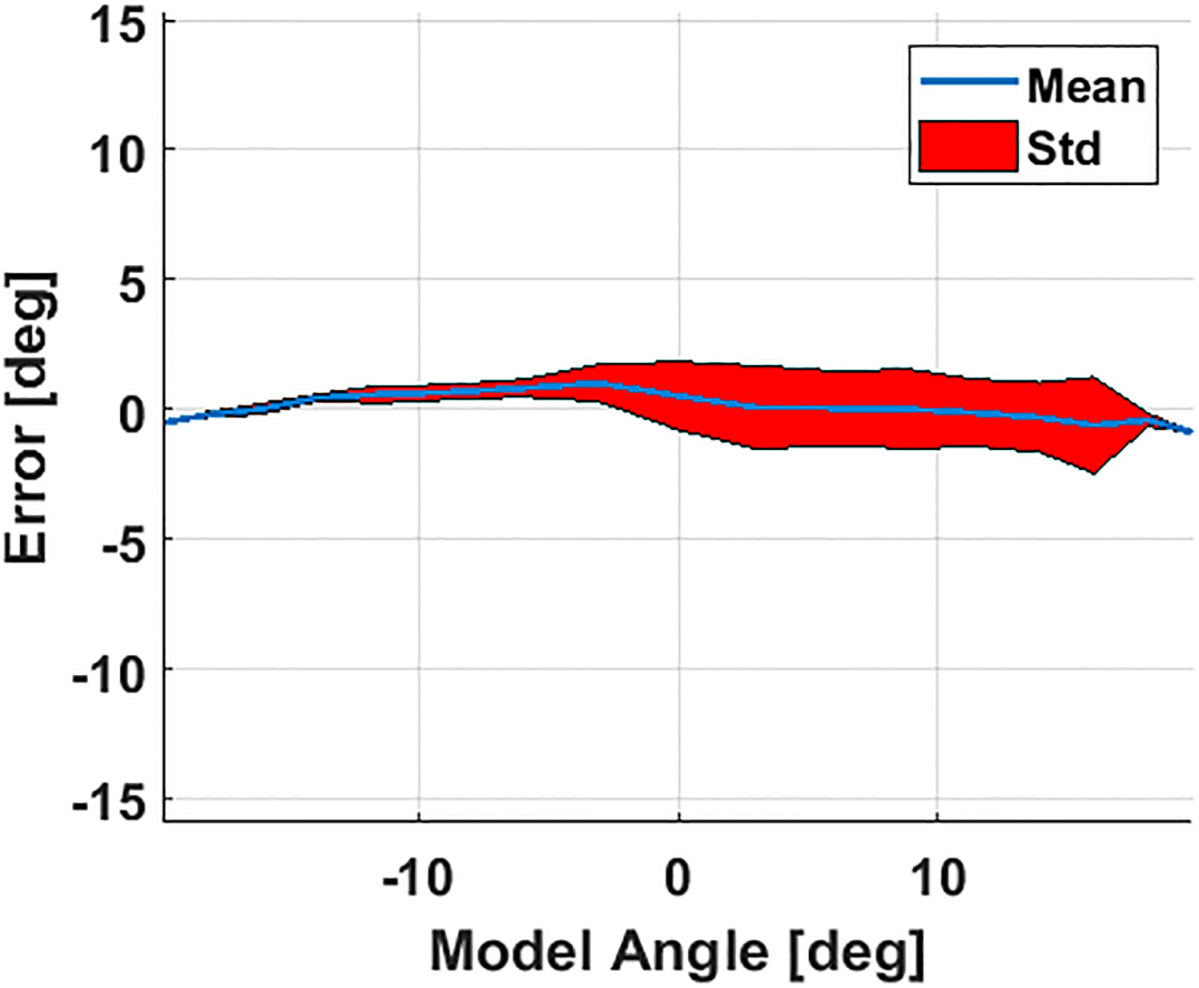
Mean and standard deviation of the error between the model and experimental results of the 1-DoF soft actuated joint (shown in their entirety in Fig 8).

Fig 8 displays both the analytical and experimental responses of the joint to combinations of pressure inputs for both rPAMs between 41 and 96 kPa [6 to 14 psi]. This figure indicates that the steady-state joint angle is a function of the pressure difference between the two rPAMs. It can be seen that the simulated and experimental results match closely, with the experimental results showing slight variations inherent to the physical world. These errors are highlighted in Fig 9, where they are divided up in terms of the model angle. We can see that the mean error is indeed small and the standard deviation is greatest when the angle is positive. Some of this error comes at the edges of the tested workspace, in particular where the pressure in the Y axis actuator (from Fig 8) was at 41 kPa (where error is around -2 degrees). One possible explanation for this discrepancy is that this actuator would buckle slightly at low pressures, causing a small increase in pressure to have a minimal effect on the joint angle. As the pressure increased more, the bend was straightened out and the actuator could provide its full force on the joint angle. This may have resulted from differences in the mounting or fabrication of each actuator.

## 4 Control strategy

Each of the two rPAMs driving the joint were connected to a 138 kPa (20 psi) pressurized air line and controlled by a separate solenoid valve. To approximate pressure inputs between 0 and 138 kPa, we operated the valves with a 30 Hz PWM signal. This resulted in a 1-DoF system being controlled by two independent inputs. Arguably, this additional control authority may be utilized to provide a common pressure value within both actuators, to control the stiffness of the overall system. In this work, to simplify joint control, we created a purely antagonistic scheme, where the binary valves are always driven in opposition to each other (*i.e. when one valve is being pressurized the opposite valve is being vented*). Thus, to control the joint angle, we specify a single number between 0 and 100 as the PWM duty cycle of one of the valves corresponding to the positive rotation of the joint, and used as the control input in the rest of this article. This method, as well as the subsequent control algorithms, was previously described in [26].

### 4.1 Sliding mode controller design based on lumped system dynamics

Using the electrical circuit equivalence of pneumatic systems from [12], we approximated the dynamic behavior of the 1-DoF joint as the following lumped second order dynamic equation:

$$\begin{array}{c} \alpha =C_{1}e^{\left(-t/\tau 1\right)}+C_{2}e^{\left(-t/\tau 2\right)}+C_{0} \end{array}$$ (11)

where *C*_1_, *C*_2_ are constant coefficients, *τ*_1_, *τ*_2_ are the time constants, and *C*_0_ is the steady-state static angle, with a one-to-one relation to the PWM duty cycle, indicating the angle the joint will converge after the dynamic terms dissipate.

**Fig 10:**
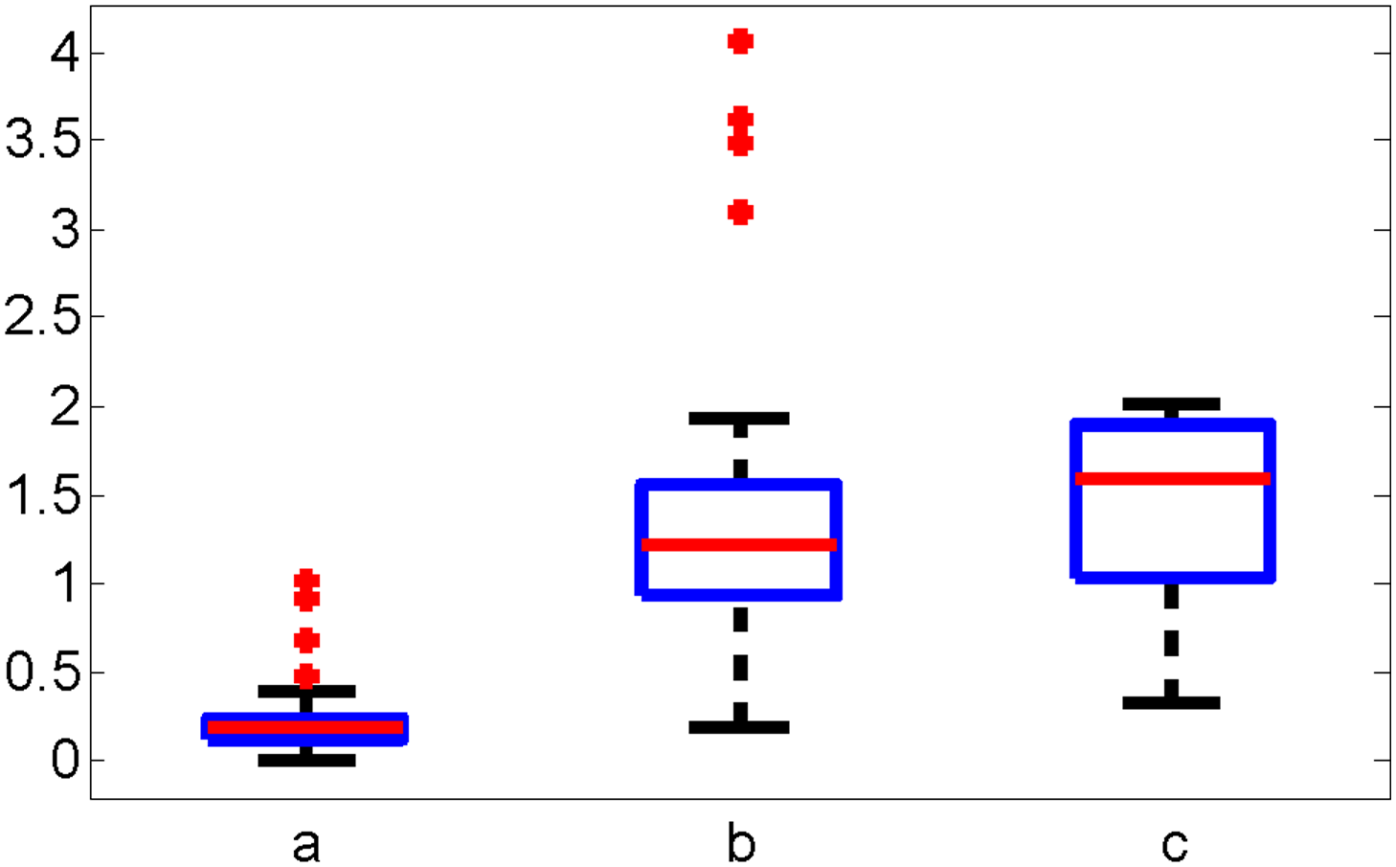
A box plot representation of the dynamic coefficients for joint response to duty cycles of 35% to 65% under 138 kPa (20 psi). The red line is the median, the blue box is the 25th and 75th percentile, the black lines are the bounds of the non-outlying data, and the red circles are the outliers. The 25th and 75th percentiles of $\hat{a},\hat{b},\hat{c}$ are (0.4123,0.3240) rad/*s*^2^, (0.9859,1.7316) rad/s and (1.2577,1.637) rad/PWM. and the median of the $\hat{a},\hat{b},\hat{c}$ is 0.2006 rad/*s*^2^, 1.2319 rad/s and 1.6022 rad/PWM.

The dynamic response of the actuator (11) can then be represented by the following second order system equation:

$$\begin{array}{c} \hat{a}\ddot{\alpha }+\hat{b}\dot{\alpha }+\alpha =\hat{c}u\left(t\right) \end{array}$$ (12)

where *α* is the rotation angle with respect to time, *u*(*t*) is the Duty Cycle of the PWM signal to the input valves and $\hat{a}\in \left(a_{o}-\Delta a,a_{o}+\Delta a\right)$, $\hat{b}\in \left(b_{o}-\Delta b,b_{o}+\Delta b\right)$ and $\hat{c}\in \left(c_{o}-\Delta c,c_{o}+\Delta c\right)$ are the bounded uncertainty parameters. *a*_*o*_, *b*_*o*_ and *c*_*o*_ and Δ*a*, Δ*b* and Δ*c* are the mean and standard deviation values for *a*, *b*, *c*. Fig 10 shows the variation of parameters $\hat{a},\hat{b}$ and $\hat{c}$ for duty cycles ranging from 35% to 65% under 138 kPa (20 psi) pressure input.

Eq (12) can be rewritten in state-space form as:

$$\begin{array}{c} \dot{x}=Ax+Bu\left(t\right) \end{array}$$ (13)

where $x={\left[\alpha ,\dot{\alpha }\right]}^{T},A=\left[\begin{array}{cc} 0 & 1\\ -\frac{1}{\hat{a}} & -\frac{\hat{b}}{\hat{a}} \end{array}\right]B=\left[0,\frac{\hat{c}}{\hat{a}}\right]$. Thus, we can design an iterative sliding mode controller for motion control based on our dynamic model and bounded uncertainty parameters. For a given reference ***x***^***ref***^, the position error is given as:

$$\begin{array}{c} e_{x}=G_{x}\left(x^{ref}-x\right), \end{array}$$ (14)

where ***G***_***x***_ = [*C*, 0]. We define a non-negative Lyapunov function candidate and its derivative as follows:

$$\begin{array}{c} V_{x}=\frac{{e_{x}}^{2}}{2}\geq 0 \end{array}$$ (15)

$$\begin{array}{c} \dot{V_{x}}=e_{x}{\dot{e}}_{x}, \end{array}$$ (16)

and select a desired dynamic error manifold as:

$$\begin{array}{c} {\dot{e}}_{x}+D_{x}e_{x}=0, \end{array}$$ (17)

where *D*_*x*_ is a positive constant. Plugging (17) into (16), we obtain a non-positive Lyapunov function derivative as:

$$\begin{array}{c} \dot{V_{x}}=-D_{x}{e_{x}}^{2}\leq 0, \end{array}$$ (18)

which will ensure stability. Combining (13) and (14),

$$\begin{array}{c} \begin{array}{cc} {\dot{e}}_{x} & =G_{x}\left({\dot{x}}^{ref}-\dot{x}\right)\\ & =G_{x}({\dot{x}}^{ref}-Ax-Bu\left(t\right))\\ & =G_{x}B\left(u_{eq}\left(t\right)-u\left(t\right)\right), \end{array} \end{array}$$ (19)

$$\begin{array}{c} G_{x}B\left(u_{eq}\left(t\right)-u\left(t\right)\right)+D_{x}e_{x}=0, \end{array}$$ (20)

where *u*_*eq*_(*t*) is the continuous equivalent control input, which is difficult to calculate [37]. Rearranging (19) reveals:

$$\begin{array}{c} u_{eq}\left(t\right)=u\left(t\right)+{\left(G_{x}B\right)}^{-1}{\dot{e}}_{x}. \end{array}$$ (21)

Approximating *u*_*eq*_(*t*) with *u*_*eq*_(*t* − Δ*t*), where Δ*t* is the time step yields the iterative sliding mode control law:

$$\begin{array}{c} u\left(t\right)=u\left(t-\Delta t\right)+{\left(G_{x}B\right)}^{-1}\left({\dot{e}}_{x}+D_{x}e_{x}\right) \end{array}$$ (22)

For simplicity, we define *K* = (***G***_***x***_ ***B***)^−**1**^ as a scalar positive tuning factor as:

$$\begin{array}{c} u\left(t\right)=u\left(t-\Delta t\right)+K\left({\dot{e}}_{x}+D_{x}e_{x}\right). \end{array}$$ (23)

### 4.2 Feed forward controller design

An improvement to this controller can be developed by taking advantage of the fact that a given control input to our system converges to a single angle at steady state. We characterized this static response by recording the resulting steady state joint angles from a range of duty cycle control inputs. Next, we mapped the solenoid valve duty cycle with respect to the resulting pressure, which we measured using a pressure sensor.

**Fig 11:**
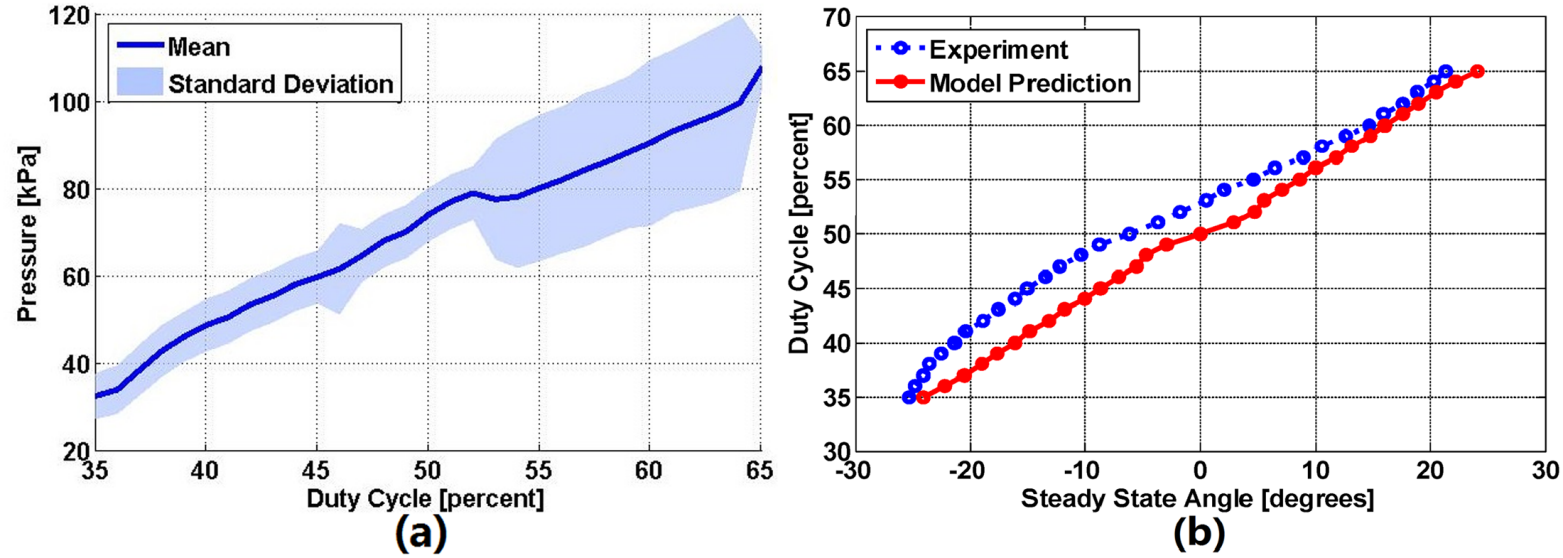
(a) The relation between the duty cycle of the solenoid valve and the resulting pressure, with an input pressure of 137 kPa (20 psi) and a pulse-width-modulation frequency of 30 Hz. (b) Steady state angle response at various duty cycles and the corresponding mapping mapping function from the experiment (Blue dotted line) and the model prediction (Red solid line).

Fig 11(a) shows the mean and standard deviation of the resulting pressure within the rPAM for a range of PWM duty cycle inputs. After characterizing this relation, we plugged it into (9) to determine the relation between the steady state angle of the joint and solenoid valve duty cycle. Fig 11(b) displays this mapping function from the model prediction (red solid line) and our experimental data (blue dotted line). The result shows that the amplitudes of the two mapping functions are close, but the experimental data displays an offset, indicating a slight bias to one direction, which is likely caused by manual fabrication differences between the two rPAMs. We fit a 3rd-order polynomial to the experimental data and incorporated it into the sliding mode controller as a feedforward term (SM+FF) as follows:

$$\begin{array}{c} u_{h}\left(t\right)=\text{Map}\left(\alpha^{ref}\right)+u\left(t\right) \end{array}$$ (24)

In this equation, *u*_*h*_(*t*) is the SM+FF output signal, *u*(*t*) is the controller signal from (23), and Map(*α*^*ref*^) is the mapped PWM duty cycle, which results in the reference angle in steady state. Substituting in, we obtain:

$$\begin{array}{c} u_{h}\left(t\right)=u\left(t-\Delta t\right)+K\left({\dot{e}}_{x}+D_{x}e_{x}\right)+\text{Map}\left(\alpha^{ref}\right) \end{array}$$ (25)

It should be noted that *u*(*t*) is still updated according to the iterative feedback law in (23), which prevents the output of the mapping function from compounding and skewing the control input.

### 4.3 Joint control results

**Fig 12:**
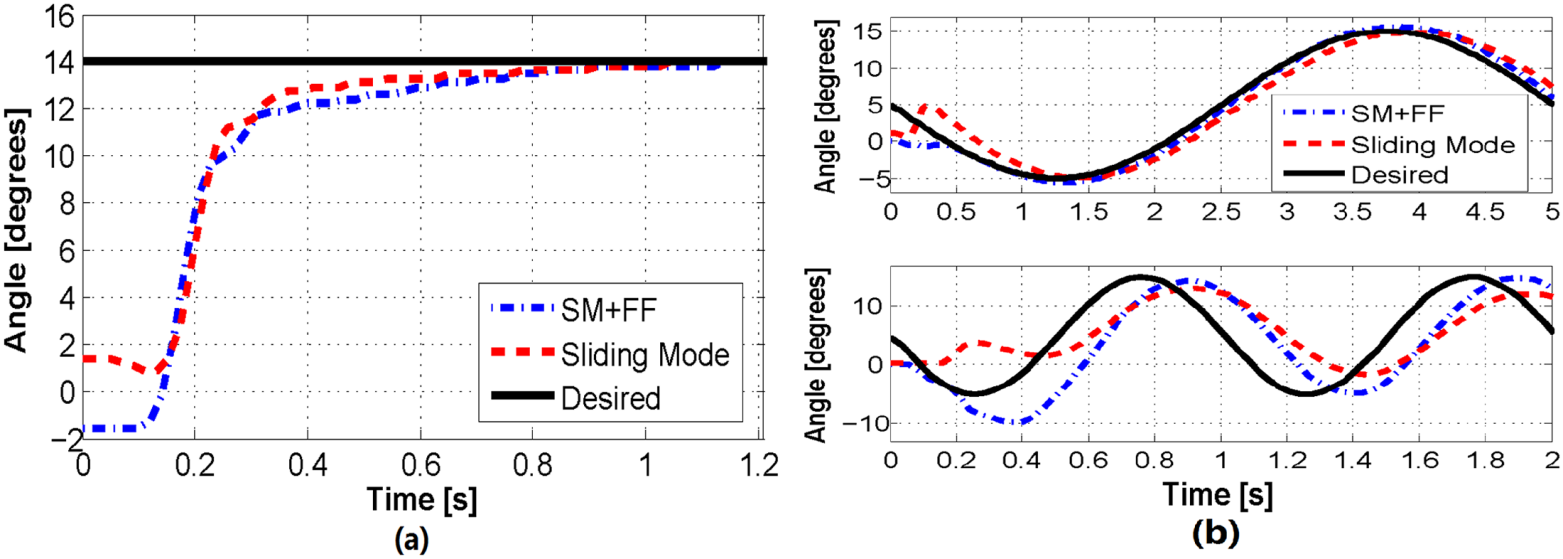
(a) A comparison of the two control algorithms on a step response. Small variations in the starting point of each trial are a result of frictional effects. (b) A comparison of the two control algorithms tracking 0.2 Hz (top) and 1 Hz (bottom) sinusoidal waves.

**Fig 13:**
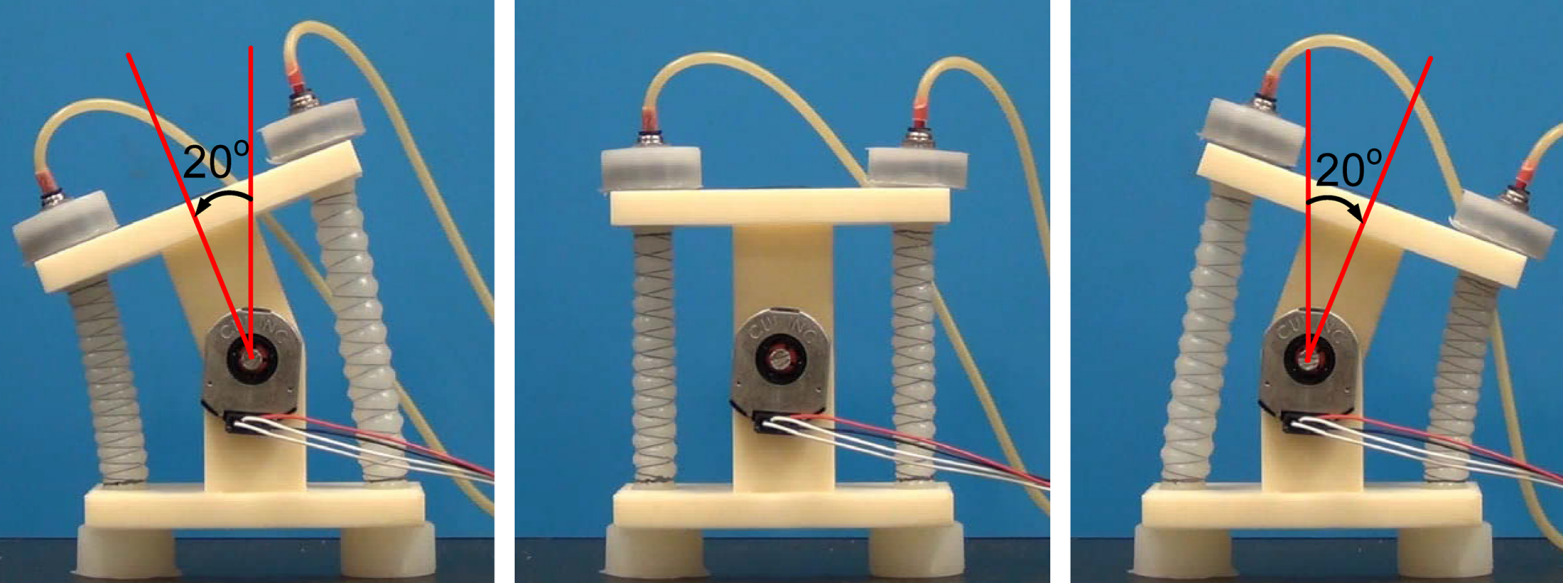
The feedforward assisted sliding mode controller offers precise position control under step reference signals in both directions.

To verify and compare the performance of our proposed feedback motion control approaches, we performed a number of experimental studies using our 1-DoF system. First, we tested the ability of our controllers to follow step reference signals, representative results of which are shown in Figs 12(a) and 13. As shown in Fig 12(a), the sliding mode and SM+FF controllers perform equally well, with a reaching time of approximately 0.8 s. The two data sets start at two slightly different angles (SM+FF at -1.5° and sliding mode at 1.5°, approximately). This is a result of friction in the joint and shifting of the actuators, which cause the neutral position of the joint to change slightly between uses. We have observed that this shift in the starting position does not effect the long-term joint trajectory. The control coefficients used for all experimental results were tuned through preliminary experiments to be *K* = 30 and *D*_*x*_ = 0.0033.

**Fig 14:**
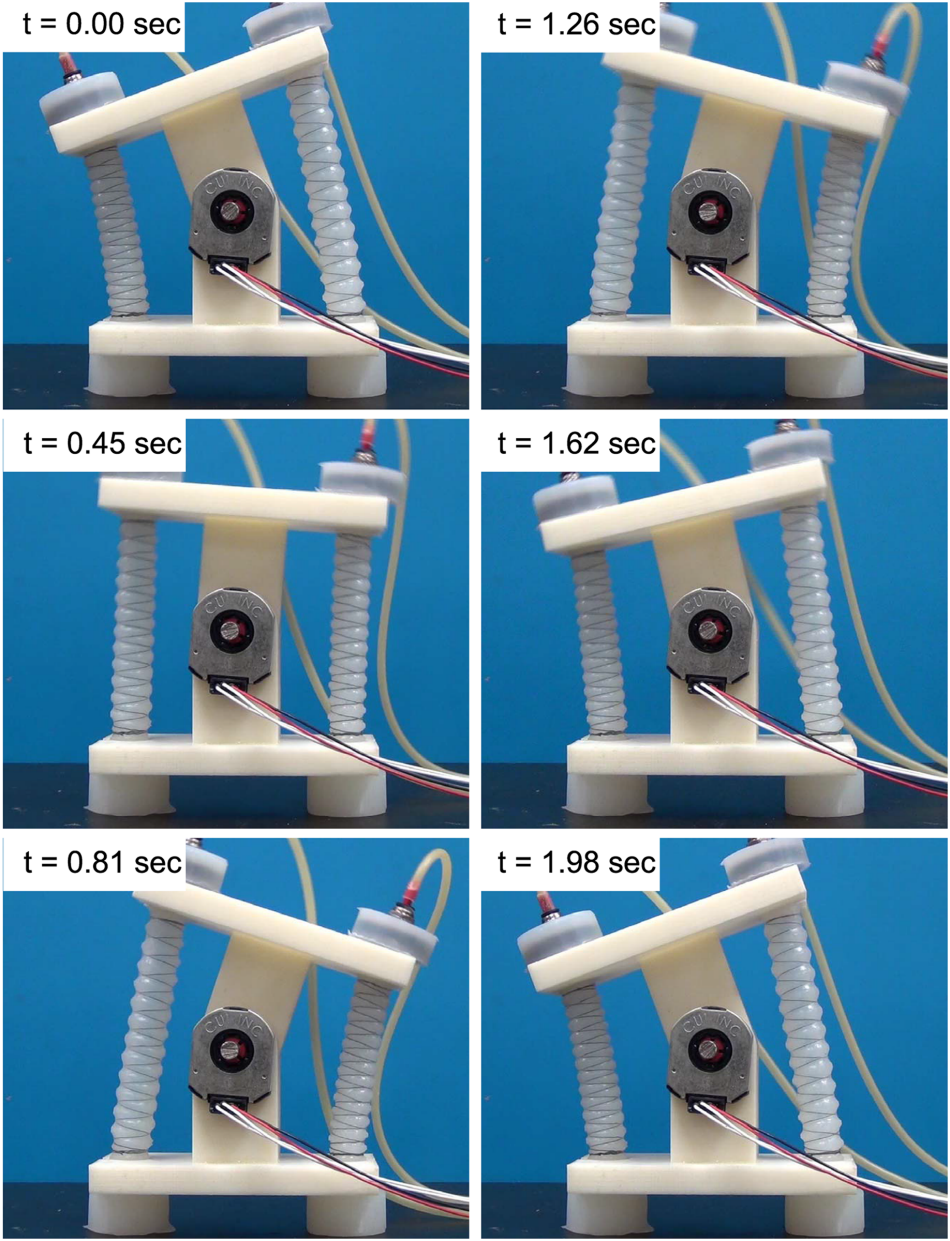
A series of snapshots taken from a 0.5 Hz sinusoidal wave tracking experiment using the SM+FF controller.

We next tested our controllers with more complicated signals: offset sinusoidal waves. Using a range of reference frequencies between 0.2 Hz and 1 Hz, we observed the performance of our controllers in a dynamic context. Experimental results of these trials can be seen in Figs 12(b) and 14. These figures indicate that the sliding mode controller follows a sinusoidal trajectory with a constant time delay with respect to the reference signal. At lower frequencies, it follows the input signal with a slight lag, while at higher frequencies it lags behind with a reduced amplitude due to the slow dynamic characteristics of the soft actuators.

The SM+FF controller follows a more unique path. It displays a similar lagging behavior to the sliding mode controller, which is a product of system delay and not knowing future changes in the trajectory. However, at lower frequencies, the SM+FF controller catches up with the input signal before each peak and overshoots slightly. This is a result of the combination of the two controllers. The FeedForward controller provides a control input capable of driving the joint to follow the signal with a delay, while the sliding mode controller works on this latent error, causing the SM+FF controller to catch up to the reference signal. Though its max amplitude is higher than the reference signal, the SM+FF response has minimal phase shift at lower frequencies, providing a level of compensation for slow actuator dynamics. At higher frequencies, the SM+FF controller can no longer catch up with the reference signal, but still follows more closely than the sliding mode controller.

**Fig 15:**
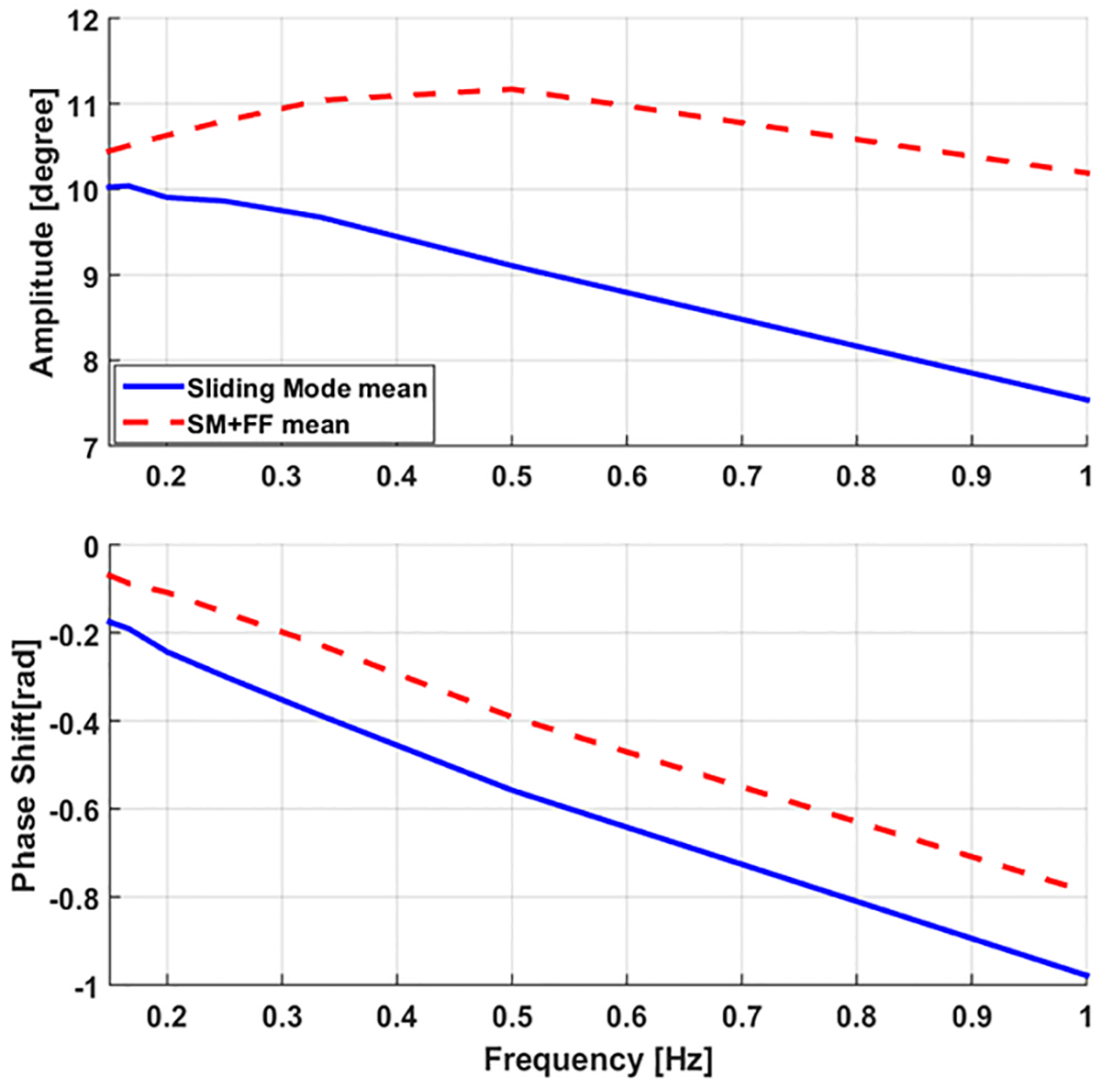
Amplitude (top) and the corresponding phase shift values (bottom) of tracking sinusoidal waveforms of 10-degree amplitude over a range of frequencies. The standard deviations for these experiments were small enough (around 0.04 degrees) not to warrant plotting, highlighting the repeatability of our joint behavior.

To confirm this observation on a wider scale, we investigated the controller responses over a series of dynamic tracking trials, each with a different frequency. Fig 15 displays a plot of the closed-loop frequency response (amplitude and phase delay) for the two controllers over the given range of sinusoidal frequencies, from 0.1 to 1 Hz. It is clear from this dataset that the SM+FF controller maintains larger amplitudes and smaller phase lags for all frequencies tested.

**Fig 16:**
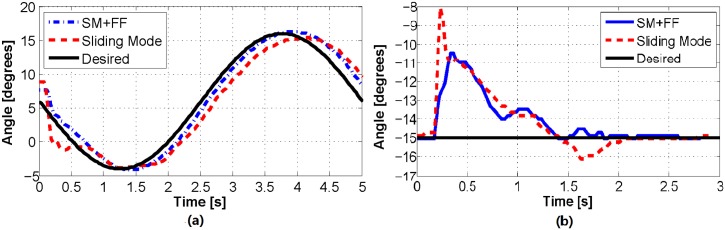
(a) A comparison of the two control algorithms following a sine wave with an external torque of 0.1 N-m acting in the positive direction. (b) Experimental comparison of the two control algorithms when a 200 g weight is added as a sudden disturbance after the step function has been reached.

We also performed the 0.2 Hz sinusoidal tracking experiment with a 200 g weight pulling perpendicular to the joint in the positive angular direction (for a total torque of approximately 0.1 N-m) to quantify the performance of the SM+FF controller when the mapping function is disturbed through external loading. The results of this experiment can be seen in Fig 16(a), which indicates that, even with the unloaded mapping function as the feedforward term, the SM+FF controller still outperforms the sliding mode feedback controller. In other words, the feedforward component of the SM+FF controller allows for an improved system response, even when the feedforward mapping function is incorrect for the tested loading case. Thus, the SM+FF controller is robust under changes to the system.

Finally, we explored the ability of the controllers to respond to a sudden disturbance, performed similarly to the previous experiment. For these experiments, we utilized a step reference signal and added the 200 g payload (applied perpendicular to the joint in the positive angular direction, as before) shortly after the set point was reached. The results, shown in Fig 16(b), indicate that the two controllers respond similarly, with a small improvement from the SM+FF controller.

## 5 Conclusion

This article represents a seamless progression from fabrication and physics-based analytical modeling to dynamic motion control of a soft pneumatic actuator we call the *reverse pneumatic artificial muscle (rPAM)*. We describe the design and fabrication of the rPAM, which is made of molded silicone rubber helically wrapped in inextensible thread. We describe numerical and analytical models of static actuator behavior and study their accuracy over a range of pressures and with external payloads up to 300 g. The models function effectively for all pressures without any external payload, but exhibit increasing error as payload increases, resulting from imprecise modeling of the constraint thread under load.

Additionally, we used a pair of rPAMs in antagonism to drive a 1-DoF revolute joint. The analytical actuator model is further extended to this joint, and its accuracy is verified through experimental studies. We also derived an iterative sliding mode feedback motion controller for this system. This controller was then combined with a static mapping function to provide feed-forward assistance to the sliding mode controller (SM+FF). The SM+FF controller was notably more effective in following a dynamic sinusoidal trajectory even under external loading and disturbance. The SM+FF and sliding mode controllers performed similarly in following a step function without external payload.

Our results show that for this application, numerical computation such as finite element method was generally not necessary for describing the rPAM behavior. The finite element method required computational time and effort, and resulted in a similar prediction to the analytical model. This is probably a result of complexity of the helical constraint, and the simplicity of the geometry which further emphasizes the value of our analytical model.

Our analytical model represents a simple and computationally efficient method of predicting soft actuator response, ideal for implementation on a micro-controller. The proposed model is modular and can easily be divided into discrete components. The material and constraint forces can be individually modified for greater fidelity or to utilize alternative material models without affecting the rest of the overall model. Additional force terms may be added as necessary (e.g. to incorporate empirical correction forces [36] or additional constraint relations).

Performance of the proposed joint was studied experimentally and analytically. It can be seen from Fig 8 that multiple pressure input combinations will result in identical joint rotation angles. The implementation of the SM+FF controller is simple, and requires minor computational complexity, since the mapping function is based on the characterization of the static response of the rPAM-driven joint under varying PWM duty cycles. For this small cost, it gains significant accuracy in dynamic signal tracking. The only shortcoming of this approach is the requirement of the initial calibration step for each new rPAM, and that potential shifts in the muscle attachment point may slightly change the static relation. The former is a relatively simple process, which can be completely automated. The latter may reduce the effectiveness of the SM+FF controller. However, it can be as seen from external loading experiments that the SM+FF maintains an improvement over the original sliding mode controller under experimental variations.

This work represents an advancement in soft pneumatic actuation. The rPAM actuators allow for fast, linear actuation. They only have significant deformation in the direction of actuation, unlike most other soft pneumatic actuators, including McKibben Muscles. This significantly increases their efficiency. Our modeling work allows for accurate predictions of the actuator behavior, reducing the trial-and-error in device design and allowing us to use optimization algorithms. The use of Pulse-Width Modulation of the analog valves allows our system to react quickly while still minimizing the necessary physical infrastructure. For example, analog pressure regulators can often have a response time of 100 ms compared to the 1.6 ms exhibited by our valves. This makes our work ideal for mobile robots or other circumstances where space is limited. The sliding mode and SM+FF control scheme allows this valve scheme to still be used for precise control, though the valve cycling reduces the ability of the system to hold steady at a specific angle.

Our current work includes refining our actuator model to compensate for the inconstancies that appear when weight is added. This could be done by refining our model to incorporate the constraint threading into *F*_*int*_ or less satisfyingly by fitting a function to the errors and adding it to the actuator force model. We are also developing more complex kinematic chains driven by multiple rPAM actuators. While this system is biologically inspired, consisting of muscle-analogs and bone-analogs, we seek to use it for more traditional robotic applications, where its safety and precision can be utilized. We are working on expanding our models predicting the behavior of more general arrangements of rPAMs, allowing them to be used to drive a robotic arm [38]. We are also investigating improvements in control algorithms to drive these kinematic chains to desired states. Future work includes further refining the proposed SM+FF controller by combining the calibrated system dynamics with the static mapping function for a more reliable dynamic FeedForward term. We also seek to improve actuator dynamics in order to achieve a higher performance to better perform tasks.

## Supporting information

S1 File Data Availability File. This file contains the data used in this paper.
(ZIP)
